# Carotid body: a new target for rescuing neural control of cardiorespiratory balance in disease

**DOI:** 10.3389/fphys.2014.00304

**Published:** 2014-08-20

**Authors:** Robert S. Fitzgerald

**Affiliations:** Departments of Environmental Health Sciences, of Physiology, and of Medicine, The Johns Hopkins Medical InstitutionsBaltimore, MD, USA

**Keywords:** chronic heart failure, cardiopulmonary, carotid body, control, removal, glomectomy

## Abstract

Significant insight into the mechanisms involved in chronic heart failure (CHF) have been provided by Schultz and his associates at the University of Nebraska Medical Center with the use of pacing-induced heart failure rabbits. Critical among the CHF mechanisms was the role of the carotid body (CB). The stimulated CB produces a wide array of systemic reflex responses; certainly those in the cardiopulmonary (CP) system are the most important in CHF. This generates a question as to whether the CB could serve as a target for some kind of treatment to reestablish control of cardiorespiratory balance in CHF. Any treatment would have to be based on a solid understanding of the mechanisms of chemosensing by the CB as well as the transducing of that sensing into neural activity sent to the medullary centers and regions of autonomic outflow to the periphery. Two avenues of treatment could be to (1) silence or attenuate the CB's neural output pharmacologically and (2) excise the CBS. There is a long history of CB removal mostly as a remedy for chronic obstructive lung disease. Results have been inconclusive as to the effectiveness of this procedure. But if carefully planned, the procedure might be a helpful treatment.

## Basic background

**Figure 1:**
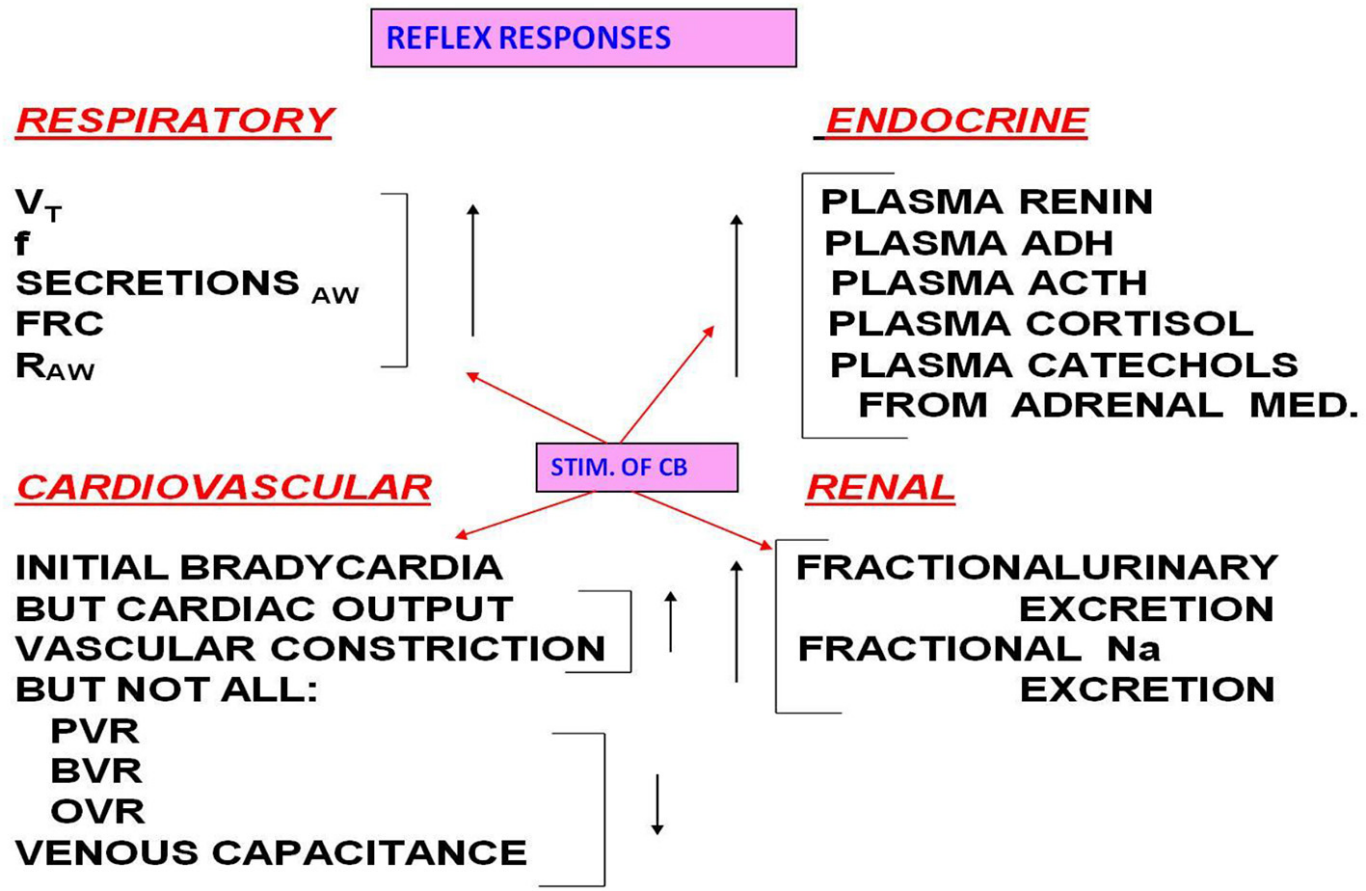
**CB stimulation increases the listed variables in the respiratory system**. Stimulation increases some CV variables but decreases pulmonary vascular resistance (PVR), bronchial vascular resistance (BVR), ocular vascular resistance (OVR), and venous capacitance. Note also the impact of CB stimulation on the endocrine and renal systems.

The stimulated carotid body (CB) provokes a wide array of cardiopulmonary (CP) reflex responses, as well as having an impact on the endocrine and renal systems (Figure 1).

This bilateral rate-sensitive interoreceptor, arguably the most essential for maintaining normal homeostasis in the organism, is located at the bifurcation of the common carotid artery into the internal carotid artery (going to the Circle of Willis in the brain) and the external carotid artery which perfuses the face and scalp. The CBs are perfused at a very high rate by a branch of the external carotid artery. Neural output from the CB is generated by excitatory neurotransmitters, released from the CB's thousands of glomus cells. They attach to receptors on abutting neurons (branches of the glossopharyngeal nerve) the cell bodies of which lie in the petrosal ganglion from which the traffic proceeds to the Nucleus Tractus Solitarii (NTS) in the brainstems's medulla. The neural traffic is increased in response to decreased partial pressure of oxygen in arterial blood(P_a_O_2_), low glucose, elevated levels of CO_2_ (P_a_CO_2_), elevated H^+^ levels [H^+^]_a_. Neural output also increases in response to increases in temperature and osmolarity.

## Activity in heart disease

Well-documented in animal models is the fact that chronic heart failure (CHF) renders the CB more sensitive (Sun et al., 1999). This increased sensitivity produces an increase in the CB's neural output even under normal acid-base conditions; part of this increase proceeds through NTS to the paramedian reticular nuclei, one seat of sympathetic neural outflow to the heart and vessels, as well as to the location of modulating serotinergic action. Increased sympathetic outflow to the ventricles is undesirable in CHF since it can provoke ventricular arrhythmias (Paterson, 2005).

## Elements of chemosensing and chemotransduction

Targeting the CBs as loci of treatment to see if homeostatic balance can be reestablished during disease requires a relatively deep understanding of the mechanisms of chemosensation and chemotransduction. In other words one must know how the CBs sense the stimuli which depolarize the glomus cells, and what are the mechanisms for converting this sensing into neural traffic.

The CB's chemosensitive structure initiating depolarization of the glomus cell is still under study though significant progress has been made.

By way of a brief overview, heme-oxygenase 2 has been proposed as the precise molecule that acts to close the calcium-sensitive K channel (aka the BK or maxi-K channel). But NADPH-oxidase and AMP-activated protein kinase have also received support for the depolarizing role. What must be kept in mind is that this important initiating molecule may not be the same in all species. For though heme-oxygenase 2 seems to function in the role in rats, one study reports that in knockout mice the absence of heme-oxygenase 2 does not prevent the hypoxia-induced release of catecholamines. That NADPH-oxidase is involved has been supported by manipulations of some of its genetic components; e.g., deletion of p47^phox^ enhanced the CB's normal responses to hypoxia.

But since the sensing of hypoxia by the CB and by the pulmonary arteries has seemed somewhat similar in the product (neural excitation and vasoconstriction), it is interesting to note that the precise O_2_ sensor in the CB remains unclear (Gonzalez et al., 1994; Lopez-Barneo, 2003). Whereas in pulmonary arterial smooth muscle cells “the bulk of evidence suggests that the primary sensor for hypoxic pulmonary vasoconstriction is the mitochondrion in the smooth muscle cells, which increases production of ROS during hypoxia… It is possible that secondary sensor mechanisms, such as ROS production by sarcolemmal NADPH oxidase, also contribute” (Sylvester et al., 2012).

K channels which are oxygen-sensitive have always been thought to play the initiating role in the depolarization of the transmitter-containing glomus cells. Additional to the K_BK_ channel, reports include other K channels as being oxygen-sensitive: TASK-like K, Kv4.1, Kv4.3 channels. These channels have always been thought to play a necessary role. The chronological steps in chemotransduction in the CBs are fairly well-known and agreed upon. Indeed, many of the sub-cellular and molecular mechanisms of sensing and transducing chemical signals have been worked out quite well, though more work needs to be done.

## Treatment of the target to reduce its impact

But on the basis of our present corpus of knowledge what can be suggested by way of treating the CB as a target for rescuing neural control of cardiorespiratory balance in disease? Two techniques suggest themselves: (a) silencing the CBs; (b) CB removal/extirpation/resection.

- Silencing (1) Dopamine is well-known to blunt the CB's neural output in response to hypoxia. (2) NO is well-known to reduce the CB's output in response to hypoxia. And a set of recent studies has shown that nNOS is reduced in CHF rabbits, a situation which is reversed by the vectoring into the CBs nNOS (Li et al., 2005). (3) A second agent reported to reduce the release of ACh and ATP, two excitatory neurotransmitters in the CBs of the cat, is Na_2_S, a precursor of H_2_S (Fitzgerald et al., 2011); this agent seems to open ATP-sensitive K channels in the cell membrane of the glomus cells. With the outflow of K^+^ ions the glomus cells become hyperpolarized, inhibiting the entry of Ca^++^ and subsequent release of neurotransmitters from the vesicles in the glomus cells. Cat CBs also tested positive for an H_2_S synthesizing enzyme, cystathionine-β-synthase. In this age of nanotechnology loading microspheres with a pharmacological agent or an enzyme and fixing a marker of some sort on the surface of the sphere which would recognize the CB does not seem overly ambitious. (4) Finally, CHF rabbits showed a reduction in CB blood flow. This condition would *per se* increase CB neural output due to the high metabolic rate of the CB lowering PO_2_ and elevating PCO_2_ in the CB. A program of regular modest exercise has been shown to be an effective way to increase CB blood flow (Li et al., 2008; Ding et al., 2011). This has been tried clinically in some hospitals, and found to be effective in attenuating symptoms of cardiac malfunction.
- Removal of the CBs could be another option. The literature addressing this option is extensive, but, regrettably, not at all conclusive. It describes results in several species. And there are different results. But human diseases for which the procedure was performed were cerebral ischemia (constricted common carotid arteries). This was treated with endarterectomy which involved CB removal. Most other reports treat CB removal as a treatment for asthma, COPD; none address CHF. Usually CB removal involves the removal of the carotid sinus sensors of blood pressure as well. If CBs are removed, do patients survive? This is, of course, the critical question. Let us review briefly a few of the more extensive studies. Nakayama (1961) used glomectomy to treat childhood asthma. Some of these patients were tested 30 years later and still exhibited no response to hypoxia. Bilateral endarterectomy in seven patients denervated carotid bodies (Wade et al., 1970) creating a permanent hypoventilation and a modest hypercapnia. In 57 cases of COPD unilateral glomectomy Phillips and Kintner (1970) concluded the procedure did not significantly alter the course of bronchospastic disease, based on a battery of pulmonary function tests 4 years post-glomectomy. On the other hand Stullberg and Winn (1989) reported an improvement in dyspnea in three men who had undergone bilateral glomectomy to offset severe COPD. All three, ages 57, 67, 69, died 6, 18, 36 months post-surgery, but remained convinced of the efficacy of the surgery even though there was no improvement in their severe airflow limitations. Whipp and Ward (1992) made a very careful quantitative study of a very large group of COPD patients the day before and the day after the surgery. No deaths were reported after a very selective removal of only the CB. Great intersubject variability was noted; but the changes in pulmonary function and blood gases were not large. Perhaps the most widely experienced investigator of bilateral carotid chemoreceptor extirpation is Yoshiyuki Honda and his colleagues. They found that exercise hyperpnea decreased in patients after the procedure (Honda et al., 1979a). In another study they found the procedure enhanced hypoxic tachycardia (Honda et al., 1988) in eight subjects 25 years post-surgery. In 11 asthmatic patients with the bilateral CB resection they reported some residual chemosensitivity some 23 years post-surgery (Honda et al., 1979b). Honda reviews these and studies in other animals (1992).

Based on the above overview the answer to the critical question is “Yes, most patients do live after CB resection.” Hence, it would seem that the procedure might be pursued. But perhaps the 1989 advice of Severinghaus in addressing bronchospastic patients might be followed (Severinghaus, 1989): They might be helped “… by permanent administration of oxygen via transcutaneous tracheal catheter. A variety of pharmacologic agents can minimize bronchospasm and infection and help clear secretions. Home oxygen concentrators and portable liquid oxygen supplies have become easily available to most patients. Only when all available methods fail to adequately relieve patients should surgical intervention be considered.” He also encourages the study of the procedure in a small group of carefully selected incapacitated patients by the NIH.

So survival after glomectomy seems to be the most frequent result, but the advantages of the procedure for better pulmonary functioning still seems to be controversial. Nevertheless, returning our focus to the advantage of glomectomy for cardiac problems, we see several more recent studies illustrating the central role of the CB's sensitivity in spontaneously hypertensive rats; the CB's discharge responses to hypoxia and hypercapnia are significantly greater than in normotensive rats (Fukuda et al., 1987). Carotid body denervation (CBD) saw no rise in young SHR animals, or a drop in blood pressure in adults (Abdala et al., 2012). Another rat study (Fletcher et al., 1992) reported how CBD eliminated the rise in blood pressure generated by chronic episodic hypoxia, such as is found in sleep apnea. Ribeiro et al. (2013) demonstrate how CBD prevents the development of insulin resistance and hypertension induced by hypercaloric diets. The most comprehensive animal study with which we are familiar is that of Marcus and his colleagues in CHF rabbits (Marcus et al., 2014). In brief, their study showed how CBD reduced sympathetic nerve activity, disordered breathing patterns, arrhythmia incidence, and sympatho-respiratory coupling in CHF rabbits. This should be considered the “gold standard” among animal studies of the effect of CBD as a focal point for rescuing neural control of cardiorespiratory balance in disease. The relevance of these studies for humans can be seen in an earlier study of patients with CHF, some of whom had normal chemosensitivity and others suffered from chemoreceptor hypersensitivity. The former group of 53 had a 3-year survival rate of 77%; the latter group of 27 had a rate of 41% (Ponikowski et al., 2001).

### Conflict of interest statement

The author declares that the research was conducted in the absence of any commercial or financial relationships that could be construed as a potential conflict of interest.

## References

[B1] Abdala A. P. McBryde F. D. Marina N. Hendy E. B. Engelman Z. J. Fudim M. (2012). Hypertension is critically dependent on the carotid body input in the spontaneously hypertensive rat. J. Physiol. 59017, 4269–4277 10.1113/jphysiol.2012.237800 22687617 PMC3473284

[B2] Ding Y. Li Y.-L. Schultz H. D. (2011). Role of blood flow in carotid body chemoreflex function in heart failure. J. Physiol. 589, 245–258 10.1113/jphysiol.2010.200584 21078591 PMC3039273

[B3] Fitzgerald R. S. Shirahata M. Chang I. Kostuk E. Kiihl S. (2011). The impact of hydrogen sulfide (H_2_S) on neurotransmitter release from the cat carotid body. Respir. Physiol. Neurobiol. 176, 80–89 10.1016/j.resp.2011.01.010 21292043 PMC3095827

[B4] Fletcher E. C. Lesske J. Behm R. Miller C. C. 3rd. Stauss H. Unger T. (1992). Carotid chemoreceptors, systemic blood pressure, and chronic episodic hypoxia mimicking sleep apnea. J. Appl. Physiol. 72, 1978–1984 1601808 10.1152/jappl.1992.72.5.1978

[B5] Fukuda Y. Sato A. Trzebski A. (1987). Carotid chemoreceptor discharge responses to hypoxia and hypercapnia in normotensive and spontaneously hypertensive rats. J. Auton. Nerv. Syst. 19, 1–84 10.1016/0165-1838(87)90139-1 3598046

[B6] Gonzalez C. Almaraz L. Obeso A. Rigual R. (1994). Carotid body chemoreceptors: From natural stimuli to sensory discharges. Physiol. Rev. 74, 829–898 7938227 10.1152/physrev.1994.74.4.829

[B7] Honda Y. (1992). Respiratory and circulatory activities in carotid body-resected humans. J. Appl. Physiol. 73, 1–8 1506355 10.1152/jappl.1992.73.1.1

[B8] Honda Y. Hashizume I. Kimura H. Severinghaus J. W. (1988). Bilateral carotid body resection in man enhances hypoxic tachycardia. Jap. J. Physiol. 38, 917–928 10.2170/jjphysiol.38.917 3249470

[B9] Honda Y. Myojo S. Hasegawa S. Hasegawa T. Severinghaus J. W. (1979a). Decreased exercise hyperpnea in patients with bilateral carotid chemoreceptor resection. J. Appl. Physiol. Respirat. Environ. Exerc. Physiol. 46, 908–912 468608 10.1152/jappl.1979.46.5.908

[B10] Honda Y. Watanabe S. Hashizume I. Satomura Y. Hata N. Sakakibara Y. (1979b). Hypoxic chemosensitivity in asthmatic patients two decades after carotid body resection. J. Appl. Physiol. Respirat. Environ. Exerc. Physiol. 46, 632–638 457538 10.1152/jappl.1979.46.4.632

[B11] Li Y. L. Ding Y. Agnew C. Schultz H. D. (2008). Exercise training improves peripheral chemoreflex function in heart failure rabbits. J. Appl. Physiol. 105, 782–790 10.1152/japplphysiol.90533.2008 18583379 PMC2536814

[B12] Li Y.-L. Li Y.-F. Liu D. Cornish K. G. Patel K. P. Zucker I. H. (2005). Gene transfer of nNOS to carotid body reverses enhanced chemoreceptor function in heart failure rabbits. Circ. Res. 97, 260–267 10.1161/01.RES.0000175722.21555.55 15994433

[B13] Lopez-Barneo J. (2003). Oxygen and glucose sensing by carotid body glomus cells. Curr. Opin. Neurobiol. 13, 493–499 10.1016/S0959-4388(03)00093-X 12965299

[B14] Marcus N. J. Del Rio R. Schultz E. P. Xia S.-H. Schultz H. D. (2014). Carotid body denervation improves autonomic and cardiac function and attenuates disordered breathing in congestive heart failure. J. Physiol. 592, 391–408 10.1113/jphysiol.2013.266221 24247985 PMC3922501

[B15] Nakayama K. (1961). Surgical removal of the carotid body for bronchial asthma. Dis. Chest 40, 595–604 10.1378/chest.40.6.595 14478244

[B16] Paterson D. J. (2005). Targeting arterial chemoreceptor over-activity in heart failure with a gas. Circ. Res. 97, 201–203 10.1161/01.RES.0000177931.10616.cb 16081873

[B17] Phillips R. M. Kintner H. P. (1970). Results of glomectomy in chronic obstructive pulmonary disease: a four year follow-up report of 57 cases. Chest 58, 358–362 10.1378/chest.58.4.358 5506630

[B18] Ponikowski P. Chua T. P. Anker S. D. Francis D. P. Doehner W. Banasiak W. (2001). Peripheral chemoreceptor hypersensitivity: an ominous sign in patients with chronic heart failure. Circulation 104, 544–549 10.1161/hc3101.093699 11479251

[B19] Ribeiro M. J. Sacramento J. F. Gonzalez C. Guarino M. P. Monteiro E. C. Conde S. V. (2013). Carotid body denervation prevents the development of insulin resistance and hypertension induced by hypercaloric diets. Diabetes 62, 2905–2916 10.2337/db12-1463 23530003 PMC3717872

[B20] Severinghaus J. W. (1989). Carotid body resection for COPD? Chest 95, 1128–1129 10.1378/chest.95.5.1128 2707069

[B21] Stullberg M. S. Winn W. R. (1989). Bilateral carotid body resection for the relief of dyspnea in severe chronic obstructive pulmonary disease. Chest 95, 1123–1128 10.1378/chest.95.5.1123 2495905

[B22] Sun S. Y. Wang W. Zucker I. H. Schultz H. D. (1999). Enhanced peripheral chemoreflex function in conscious rabbits with pacing-induced heart failure. J. Appl. Physiol. 86, 1264–1272 10194212 10.1152/jappl.1999.86.4.1264

[B23] Sylvester J. T. Shimoda L. A. Aaronson P. I. Ward J. P. T. (2012). Hypoxic pulmonary vasoconstriction. Physiol. Rev. 92, 367–520 10.1152/physrev.00041.2010 22298659 PMC9469196

[B24] Wade J. G. Larson C. P. Jr. Hickey R. F. Ehrenfeld W. K. Severinghaus J. W. (1970). Effect of carotid endarterectomy on carotid chemoreceptor and baroreceptor function in man. N. Engl. J. Med. 282, 823–829 10.1056/NEJM197004092821501 5418544

[B25] Whipp B. J. Ward S. A. (1992). Physiologic changes following bilateral carotid body resection in patients with chronic obstructive pulmonary disease. Chest 101, 656–661 10.1378/chest.101.3.656 1541128

